# Nosocomial Pandemic (H1N1) 2009, United Kingdom, 2009–2010

**DOI:** 10.3201/eid1704.101679

**Published:** 2011-04

**Authors:** Joanne E. Enstone, Puja R. Myles, Peter J.M. Openshaw, Elaine M. Gadd, Wei Shen Lim, Malcolm G. Semple, Robert C. Read, Bruce L. Taylor, James McMenamin, Colin Armstrong, Barbara Bannister, Karl G. Nicholson, Jonathan S. Nguyen-Van-Tam

**Affiliations:** Author affiliations: University of Nottingham, Nottingham, UK (J.E. Enstone, P.R. Myles, J.S. Nguyen-Van-Tam);; Imperial College, London, UK (P.J.M. Openshaw);; Department of Health, London (E.M. Gadd, C. Armstrong, B. Bannister);; Nottingham University Hospitals National Health Service Trust, Nottingham (W.S. Lim);; University of Liverpool, Liverpool, UK (M.G. Semple);; University of Sheffield, Royal Hallamshire Hospital, Sheffield, UK (R.C. Read);; Portsmouth Hospitals National Health Service Trust, Portsmouth, UK (B.L. Taylor);; Health Protection Scotland, Glasgow, Scotland (J. McMenamin);; University Hospitals of Leicester National Health Service Trust, Leicester, UK (K.G. Nicholson)

**Keywords:** Nosocomial infections, influenza, pandemic (H1N1) 2009, influenza A virus, infection control, hospitalization, viruses, United Kingdom, expedited, research

## Abstract

To determine clinical characteristics of patients hospitalized in the United Kingdom with pandemic (H1N1) 2009, we studied 1,520 patients in 75 National Health Service hospitals. We characterized patients who acquired influenza nosocomially during the pandemic (H1N1) 2009 outbreak. Of 30 patients, 12 (80%) of 15 adults and 14 (93%) of 15 children had serious underlying illnesses. Only 12 (57%) of 21 patients who received antiviral therapy did so within 48 hours after symptom onset, but 53% needed escalated care or mechanical ventilation; 8 (27%) of 30 died. Despite national guidelines and standardized infection control procedures, nosocomial transmission remains a problem when influenza is prevalent. Health care workers should be routinely offered influenza vaccine, and vaccination should be prioritized for all patients at high risk. Staff should remain alert to the possibility of influenza in patients with complex clinical problems and be ready to institute antiviral therapy while awaiting diagnosis during influenza outbreaks.

Nosocomial influenza is a well-recognized problem in acute-care hospital settings (*1**,**2*). Outbreaks of influenza A have been reported in general wards (*3**,**4*), pediatric units (*5*), neonatal intensive care units (ICUs) (*6**–**8*), hemopoietic and solid organ transplantation units (*9**–**11*), oncology and neurology units (*12**,**13*), and facilities for the elderly and for long-term care (*14**–**17*). Associated illness and death rates are particularly high in immunocompromised patients (*18**–**20*).

On June 11, 2009, the World Health Organization reported the first influenza pandemic of the 21st century (*21**,**22*). Although most cases of pandemic (H1N1) 2009 have been mild or subclinical, patients with severe disease have considerably affected hospital systems (*23*). Three nosocomial outbreaks of pandemic (H1N1) 2009 were reported in hemopoietic transplantation units and oncology wards. One outbreak was reportedly mild (*24*), and the other 2 involved aggressive illness, severe complications, and deaths (*25**,**26*).

In addition to outbreaks of nosocomial influenza, sporadic nosocomial influenza infections also occur but generally are not reported in the literature. We describe the clinical and epidemiologic characteristics of nosocomial pandemic (H1N1) 2009 infections during 2009–2010 in the United Kingdom that were identified during surveillance rather than through outbreak control activity.

## Methods

During the pandemic (H1N1) 2009 outbreak in the United Kingdom, the Influenza Clinical Information Network (FLU-CIN) collected clinical and epidemiologic data for patients with virologically confirmed pandemic (H1N1) 2009 virus infection admitted to hospitals (*27*). Data included demography, symptoms, medical history, influenza vaccination history, relevant timelines, investigations and results, treatment (e.g., antiviral and antibacterial drugs), outcome, and cause of death when available. Trained health care workers abstracted data from case notes. During May 11, 2009–January 31, 2010, data were accrued from 75 National Health Service hospitals in 31 cities or towns in England, Scotland, Wales, and Northern Ireland.

From this source cohort, we defined patients with nosocomial pandemic (H1N1) 2009 as those admitted to a hospital for a reason other than acute respiratory infection in whom respiratory symptoms developed >72 hours (3 days) after admission. In addition, we included infants who had not left the hospital since birth in whom pandemic (H1N1) 2009 had developed. We included transfers from other hospitals when a transfer was for a reason other than influenza and when the history of influenza clearly indicated that it had been acquired at another hospital. FLU-CIN procedures were reviewed and approved by the Ethics and Confidentiality Committee of the National Information Governance Board for Health and Social Care in England for collection, storage, and use of personal data for surveillance purposes.

## Results

Of 1,520 patients in the FLU-CIN cohort, illnesses in 30 (2.0%) (15 children) met the criteria for nosocomial influenza (Tables 1, 2 [adults] and Tables 3, 4 [children]). Patient ages ranged from 41 days to 76 years at onset of influenza symptoms (median age 44 years for adults and 1 year for children).

**Table 1:** Characteristics of 15 hospitalized adults with nosocomial pandemic (H1N1) 2009, United Kingdom, 2009–2010

Patient no.	Age, y/sex	Reason for admission	Main underlying illnesses	Signs and symptoms
1	51/F	Pancreatitis	None recorded	Fever, unknown data in other fields
2	44/M	Transplant complications	Lymphoma	Productive cough, headache, coryza, myalgia
3	34/M	Emergency surgery	Diabetes	Unknown
4	18/F	Elective surgery	Neurodegenerative disease	Dyspnea, malaise
5	48/M	Chemotherapy	Hematologic malignancy	Fever, sore throat
6	43/M	Pancreatitis	Chronic liver disease	Fever, malaise, myalgia
7	51/M	Not recorded	Lymphoma	Fever, dry cough, diarrhea, myalgia, arthralgia
8	39/F	Metastatic soft tissue	Malignancy	Dry cough, dyspnea
9	76/M	Elective surgery	Diabetes, heart disease	Productive cough, diarrhea, dyspnea
10	45/F	Not stated	Myeloma	Fever, productive cough, nausea, anorexia, malaise
11	44/F	Psoriasis	Psoriasis	Fever, unknown data in other fields
12	22/M	Posttransplant complications	Renal transplant, congenital abnormalities	Fever, cough, anorexia, malaise
13	52/M	Elective procedure	Lymphocytic leukemia	Dyspnea, altered consciousness
14	33/F	Obstetric complications	None recorded	Fever, productive cough
15	60/M	Cerebrovascular disease	Diabetes, obesity	Unknown

**Table 2:** Timelines and outcomes for 15 hospitalized adults with nosocomial pandemic (H1N1) 2009, United Kingdom, 2009–2010

Patient no.	Age, y/sex	Duration, d		Maximum level of care*	Outcome†
		Hospital admission to symptom onset	Symptom onset to receipt of antiviral therapy		
1	51/F	26	0	0/1	Unknown data
2	44/M	14	0	0/1	Recovered
3	34/M	8	0	3	Died
4	18/F	4	Not given	2	Transferred to other hospital
5	48/M	9	4	0/1	Recovered
6	43/M	5	0	0/1	Recovered
7	51/M	29	0	3	Died
8	39/F	5	3	0/1	Died
9	76/M	11	Not given	3	Died
10	45/F	24	2	0/1	Recovered
11	44/F	14	Not given	3	Transferred, improved
12	22/M	5	0	3	Died
13	52/M	78	1‡	3	Recovered
14	33/F	7	3	0/1	Recovered
15	60/M	13	Not given	3	Recovered

*Level 0 care is given to patients whose care needs can be met through normal ward care. Level 1 care is given to patients at risk for a deteriorating condition or recently relocated from higher levels of care whose needs can be met in an acute-care ward with additional advice and support from the critical-care team. Level 3 care is given to patients requiring advanced respiratory support alone or basic respiratory support and support for >2 organ systems; this level includes all patients with complex conditions that required support for multiorgan failure (intensive care unit). Level 2 care is given to patients requiring more detailed observation or intervention, including support for a single failing organ system and those changing from higher levels of care (high dependency unit). †Deaths were attributed to pandemic (H1N1) 2009. ‡Oseltamivir was replaced with zanamivir on day 5 because of identification of the H275Y drug-resistance mutation.

**Table 3:** Characteristics of 15 hospitalized children with nosocomial pandemic (H1N1) 2009, United Kingdom, 2009–2010

Patient no.	Age/sex	Reason for admission	Main underlying illnesses	Signs and symptoms
16	12 y/F	Elective surgery	Heart disease	Fever, unknown data in other fields
17	2 y/M	Malignancy	Malignancy	Dry cough, coryza
18	4 y/F	Bone marrow aspirate	Acute myeloid leukemia	Fever, productive cough
19	15 y/M	Ulcerative colitis	Ulcerative colitis	Fever, unknown data in other fields
20	123 d/F	Inpatient care from birth	Prematurity	Fever, dyspnea
21	1 y/F	Laryngomalacia, transfer from tertiary care center	Genetic disorder	Fever, dyspnea
22	1 y/M	Investigation	Acute lymphoblastic leukemia	Fever, dry cough, coryza
23	9 y/M	Sepsis	Cerebral palsy, septic pressure sore	Fever, patient sedated and ventilated
24	12 y/M	Anorexia	Anorexia	Fever, coryza, nausea, sneezing
25	82 d/M	Inpatient care from birth	Congenital abnormalities	Coryza, dyspnoea
26	64 d/M	Inpatient care from birth	Congenital abnormalities	Fever, coryza
27	151 d/M	Prematurity	Prematurity	Fever, coryza
28	101 d/F	Inpatient care from birth	Congenital abnormalities	Fever, coryza
29	41 d/F	Inpatient care from birth	Cystic fibrosis	Fever, rash
30	9 y/F	Elective surgery	Hematologic malignancy	Fever

**Table 4:** Timelines and outcome for 15 hospitalized children with nosocomial pandemic (H1N1) 2009, United Kingdom, 2009–2010

Patient no.	Age/sex	Duration, d		Maximum level of care*	Outcome
		Hospital admission to symptom onset	Symptom onset to receipt of antiviral therapy		
16	12 y/F	10	Not given	3	Recovered
17	2 y/M	24	Not given	0/1	Died at home
18	4 y/F	54	2†	0/1	Recovered
19	15 y/M	11	8	0/1	Recovered
20	123 d/F	123‡	Unknown data	3	Died after transfer to another hospital
21	1 y/F	14	1	0/1	Recovered
22	1 y/M	6	5	3	Recovered
23	9 y/M	Unknown (transferred)	3	3	Recovered
24	12 y/M	14	Not given	0/1	Recovered
25	82 d/M	82‡	1	3	Died§
26	64 d/M	64‡	Not given	1	Recovered
27	151 d/M	151‡	3	3	Recovered
28	101 d/F	101‡	1	3	Recovered
29	41 d/F	41‡	1	3	Recovered
30	9 y/F	12	1	0/1	Recovered

*Level 3 care is given to patients requiring advanced respiratory support alone or basic respiratory support and support for >2 organ systems; this level includes all patients with complex conditions that required support for multiorgan failure (intensive care unit). Level 0 care is given to patients whose care needs can be met through normal ward care. Level 1 care is given to patients at risk for a deteriorating condition or recently relocated from higher levels of care whose needs can be met in an acute-care ward with additional advice and support from the critical-care team. †Oseltamivir was replaced with zanamivir on day 11 because of identification of the H275Y drug-resistant mutation (patient also received acyclovir throughout hospitalization); ‡Inpatient since birth. §Attributed to pandemic (H1N1) 2009.

### Concurrent Conditions and Reasons for Admission

Twelve (80%) adults and 14 (93%) children had serious underlying illnesses. The most common illnesses were hematologic malignancy for adults (5), and congenital abnormality or prematurity (7) or malignancy (4) for children.

Of the 15 adults, 2 had been admitted for elective surgical procedures; 1 for emergency surgery; and 8 for deterioration of chronic conditions, including complications caused by chemotherapy, malignancy, or transplantation. Two patients were admitted for pancreatitis (1 of whom had underlying myeloma); 1 patient was admitted for obstetric complications, and another patient was admitted for psoriasis. Of 15 children, 3 were admitted for elective procedures, 6 had been in the hospital since birth (because of prematurity or congenital abnormality), 1 was transferred from another hospital, and 5 had acute conditions (Table 2).

### Pandemic Vaccination Status and Use of Antiviral and Antibacterial Drugs

None of the patients had received pandemic influenza vaccine. Although 14 adults were eligible because of concurrent conditions, influenza symptoms developed in 11 either before vaccine became available or before they would have seroconverted if vaccinated at the earliest opportunity (vaccine became available in the United Kingdom at the end of October 2009). Four children were eligible because of age and concurrent conditions, and symptoms developed in 3 before vaccine became available or before they would have seroconverted. Only 2 patients (both adults) had received seasonal influenza vaccine.

Twenty-one (72%) of 29 patients (10 children) received antiviral medication as inpatients (data were unknown for 1 patient); all initially received oseltamivir as monotherapy. Therapy for 2 patients (nos. 13 and 18) was switched from oseltamivir to zanamivir after 4 days and 10 days, respectively, because drug-resistant virus carrying the H275Y mutation was identified. Administration of antiviral drugs ranged from 0 to 8 days after symptom onset; 12 (57%) of 21 patients who received therapy did so within 48 hours. Sixteen patients were already receiving antibacterial drugs when influenza symptoms began. Two of these patients had a bacterial co-infection: coagulase-negative staphylococci in a blood culture for 1 patient and *Pseudomonas aeruginosa* in an unspecified intravenous line in 1 patient. Twelve patients received antibacterial drugs during their respiratory illness, 2 of whom had *Haemophilus influenzae* in sputum samples and 1 (co-infected with rhinovirus) who had had a blood culture positive for *Klebsiella* sp.

### Signs and Symptoms

The most common signs were fever (8 [53%] adults and 12 [80%] children), cough (10, mostly adults), coryza (8, mostly children), and dyspnea (7). Fewer patients had malaise (4); myalgia (3); anorexia, nausea, diarrhea (2 each); and arthralgia, sore throat, headache, vomiting, altered consciousness, sneezing, and rash (a child) (1 each).

### Course of Illness

Median length of hospitalization before onset of influenza symptoms was 11 days for adults (range 4–78 days) and 13 days (range 6–54 days) for children, excluding infants in a hospital since birth. For infants in a hospital since birth, the interval from birth to onset of influenza signs ranged from 41 to 123 days (median 78 days).

Results of chest radiography ≤3 days after onset of influenza symptoms were documented for 8 adults and 5 children (43%). Of these patients, 4 adults and 1 child (38%) had radiologically confirmed pneumonia.

Level 0 is care given to patients whose care needs can be met through normal ward care. Level 1 care is given to patients at risk for a deteriorating condition or recently relocated from higher levels of care whose needs can be met in an acute-care ward with additional advice and support from the critical-care team. Level 2 care is given to patients requiring more detailed observation or intervention, including support for a single failing organ system and those changing from higher levels of care (high dependency unit). Level 3 care is given to patients requiring advanced respiratory support alone or basic respiratory support and support for >2 organ systems. This level includes all patients with complex conditions requiring support for multiorgan failure (ICU).

Seven adults and 8 children (50%) required level 3 care (ICU, pediatric ICU, or neonatal ICU). One (3%) adult required level 2 care. Six adults required mechanical ventilation and 1 required noninvasive ventilation (data for ventilatory support were unknown for 1 adult). Three children required mechanical ventilation and 1 required noninvasive ventilation. The remaining 4 children who received level 3 care were 3 infants and 1 child, each of whom required a period of close monitoring, but did not ultimately require ventilation. The remaining 7 adults and 7 children required level 0 or 1 care.

### Outcomes and Mortality Rates

Five (33%) of 15 adults died in the acute-care hospital that provided treatment, 2 within 30 days after symptom onset. Of adults who died, 3 had underlying malignancy (1 noted to be terminal) or were immunocompromised and 2 had diabetes (type I and type II respectively). Pandemic (H1N1) 2009 was included in the recorded causes of death for all 5 adults. Although some patients had a prolonged hospital stay of <7 months, all remaining adults recovered from influenza and were discharged from the hospital.

Of 15 children, 3 (20%) were known to have died, although only 1 (a neonate with multiple congenital problems) died at the hospital where surveillance was conducted; acute respiratory distress syndrome/lower respiratory tract infection was stated as a cause of death. Another child, with malignancy, whose death was expected, died at home shortly after discharge. The third child was transferred to another hospital, and cause of death is unknown. All other children recovered from pandemic (H1N1) 2009. Two children remained in the hospital for treatment of their underlying malignancy, and the other children were discharged.

## Discussion

Although pandemic (H1N1) 2009 produced a generally mild illness, in the United Kingdom, as elsewhere, severe illness developed in a small proportion of relatively young patients who required hospitalization (*28*). Although nosocomial outbreaks of pandemic (H1N1) 2009 have been described (*24**–**26*), sporadic nosocomial cases of pandemic (H1N1) 2009 identified during surveillance activities have not been described. The present case series has the advantage of being derived from a larger cohort of hospital inpatients in whom confirmation of pandemic (H1N1) 2009 was obtained by using nationally standardized PCR criteria, from settings where clinical management and infection control precautions were driven by national guidelines (*29**,**30*), and with data abstracted by trained nurses (*27*).

We based our definition of nosocomial influenza on a recent study of health care–associated influenza in children (*31*). A recent systematic review of incubation periods of acute respiratory viral infections found that the median incubation period for influenza was 1.4 days for influenza A, and symptoms developed in 95% of patients in <2.8 days (*32*). These findings suggest that our cutoff point, 3 days after admission, make inclusion of community cases unlikely. In addition, in no patients did onset of respiratory illness occur <4 days after hospital admission; median length of hospitalization before symptom onset was 11 days for adults and 13 days for infants. Therefore, inadvertent inclusion of community-acquired cases is highly unlikely.

On the basis of information obtained in the study, we cannot determine where and from whom patients acquired influenza. However, 3 routes are possible. First, infection could have been acquired from other patients; 1 patient shared a bay with a patient who was presymptomatic at the time but for whom influenza was diagnosed 1 day later. Second, transmission from visitors of patients cannot be ruled out. Although national guidelines strongly discourage persons with influenza-like symptoms from visiting patients (*29*), this recommendation may have been difficult to implement, particularly for parents of sick children who often provide most hands-on care in a hospital. Third, transmission may have occurred from an infectious health care worker (because staff continue to work when infected with influenza [*33*]) or from contaminated hands of a health care worker. Transmission from asymptomatic persons might occur in all 3 instances (*34*).

Nosocomial cases in this study occurred equally in adults and children. Consistent with previous findings (*3*), most patients had >1 serious underlying illnesses, notably hematologic malignancies, congenital disorders, or prematurity. Staff and caregivers of patients with hematologic malignancy and prematurity are often particularly vigilant for symptoms suggestive of infectious disease. Although we detected nosocomial influenza in patients admitted to nonmedical areas (for emergency or elective surgery), many cases of nosocomial infection in other patient groups probably have been overlooked, particularly because influenza in these groups is likely to have been milder. Additionally, some patients are likely to have been discharged from a hospital during the incubation period of nosocomially acquired pandemic influenza. Thus, in this case series, detecting such patients would not have been possible.

More than half of patients required level 2 or level 3 care, which is higher than that required by the source cohort (12%) (*27*). Approximately one fifth of children and one third of adults died. Although the deaths of 2 patients were expected because of the stage of their underlying malignancy, this case-fatality rate is far higher than that for patients with pandemic (H1N1) 2009 and concurrent conditions in the source cohort (5%) (*27*). The combined factors of increased host susceptibility (*18**–**20*), prolonged virus shedding in immunocompromised children (*35*), and increased likelihood of development of drug resistance (*36*) raise questions about the need for enhanced infection control procedures in special-care–infant units, pediatric wards, and hemopoietic transplant units and a requirement that staff working in these areas be vaccinated (*37**–**39*). Precautions should include restricting unnecessary movement of patients to units with particularly vulnerable patients and postponement of semi-elective (nonurgent) procedures for hematology patients during peak pandemic activity.

Vaccine against pandemic (H1N1) 2009 became available at the end of October 2009. Assuming a 2-week period for vaccine administration, case-patients in groups at risk with influenza onset dates after November 30, 2009, could have been vaccinated and would have had time to seroconvert (14 days). Using these criteria, we determined that 4 cases (in 3 adults and 1 child) (13%) were potentially preventable by vaccination; 2 of these patients required escalated care, and 1 patient died. Although 72% of patients received antiviral therapy, similar to 75% in the source cohort (*27*), we observed avoidable delays between recording of respiratory symptoms and start of specific antiviral therapy in some adults and all children. Although under ordinary circumstances the complex clinical picture of such patients might result in delayed or incidental finding of influenza, in a pandemic situation or during a seasonal epidemic clinicians should be alert to the possibility of influenza. Delays encountered in this series most likely reflect a failure to consider such a diagnosis early. Other reasons are caution or uncertainty in using oseltamivir in patients younger than the drug licensing permits (12 months) in nonpandemic situations, reluctance to empirically instigate antiviral treatment in advance of a confirmed diagnosis of pandemic (H1N1) 2009, lack of confidence about absorption of oseltamivir by nasogastric tube insertion in patients already receiving mechanical ventilation or concerns about potential gastrointestinal side effects.

Nosocomial infections with pandemic (H1N1) 2009 in this case series were associated with high rates of illness and death. This finding highlights the need for adherence to infection control guidelines for staff and visitors (including the need to urge visitors not to visit when they are ill, particularly when providing hands-on care for vulnerable children), staff vaccination, maintenance of clinical suspicion for influenza in areas of high risk, prompt (empirical) antiviral treatment for vulnerable patients in whom influenza is possible or likely, and consideration of postponing nonurgent procedures for hematology patients during periods of known high influenza activity. This report demonstrates that nosocomial transmission is a recurrent problem when the prevalence of influenza is high and the total effect of nosocomial influenza is underestimated by outbreak reports alone.
